# Looking at Thyroid Cancer from the Tumor-Suppressor Genes Point of View

**DOI:** 10.3390/cancers14102461

**Published:** 2022-05-17

**Authors:** Sadegh Rajabi, Catherine Alix-Panabières, Arshia Sharbatdar Alaei, Raziyeh Abooshahab, Heewa Shakib, Mohammad Reza Ashrafi

**Affiliations:** 1 Traditional Medicine and Materia Medica Research Center, Shahid Beheshti University of Medical Sciences, Tehran 19839-63113, Iran; sadegh.rajabi2017@gmail.com; 2 Department of Clinical Biochemistry, School of Medicine, Shahid Beheshti University of Medical Sciences, Tehran 19839-63113, Iran; 3 Laboratory of Rare Human Circulating Cells (LCCRH), University Medical Centre of Montpellier, CEDEX 5, 34093 Montpellier, France; 4 Centre for Ecological and Evolutionary Cancer Research (CREEC), Unité Mixte de Recherches, Institut de Recherche pour le Développement (IRD) 224–Centre National de Recherche Scientifique (CNRS) 5290–University of Montpellier, 34000 Montpellier, France; 5 Department of Medical Genetics, Shahid Beheshti University of Medical Sciences, Tehran 19839-63113, Iran; m.alaei@icloud.com; 6 Curtin Medical School, Curtin University, Bentley, WA 6102, Australia; ra_shahab@yahoo.com; 7 Cellular and Molecular Endocrine Research Center, Research Institute for Endocrine Sciences, Shahid Beheshti University of Medical Sciences, Tehran 19857-17443, Iran; shakib.hiva@gmail.com; 8 Department of Biochemistry, Afzalipoor Faculty of Medicine, Kerman University of Medical Sciences, Kerman 76169-13555, Iran; aarshaam4000@gmail.com

**Keywords:** thyroid cancer, tumor-suppressor gene, inactivation, mutation, gene therapy

## Abstract

**Simple Summary:**

Thyroid cancer is the most common endocrine cancer. As tumor-suppressor genes (TSGs) are implicated in many different functions in the organism, their loss in cells in a normal tissue may drive their transformation into cancer cells. TSGs are generally classified into three subclasses: (i) gatekeepers that encode proteins involved in the control of cell cycle and apoptosis; (ii) caretakers that produce proteins implicated in maintaining genomic stability; and (iii) landscapers that, when mutated, create a suitable environment for neoplastic growth. Different inactivation mechanisms may suppress TSG function. Understanding these mechanisms and TSG alterations in thyroid tumors is of great importance for thyroid cancer prognosis, diagnosis, and therapy. The present review paper discusses TSG inactivation mechanisms and alterations in order to help to identify more efficient therapeutic modalities for thyroid cancer management.

**Abstract:**

Thyroid cancer is the most frequent endocrine malignancy and accounts for approximately 1% of all diagnosed cancers. A variety of mechanisms are involved in the transformation of a normal tissue into a malignant one. Loss of tumor-suppressor gene (TSG) function is one of these mechanisms. The normal functions of TSGs include cell proliferation and differentiation control, genomic integrity maintenance, DNA damage repair, and signaling pathway regulation. TSGs are generally classified into three subclasses: (i) gatekeepers that encode proteins involved in cell cycle and apoptosis control; (ii) caretakers that produce proteins implicated in the genomic stability maintenance; and (iii) landscapers that, when mutated, create a suitable environment for malignant cell growth. Several possible mechanisms have been implicated in TSG inactivation. Reviewing the various TSG alteration types detected in thyroid cancers may help researchers to better understand the TSG defects implicated in the development/progression of this cancer type and to find potential targets for prognostic, predictive, diagnostic, and therapeutic purposes. Hence, the main purposes of this review article are to describe the various TSG inactivation mechanisms and alterations in human thyroid cancer, and the current therapeutic options for targeting TSGs in thyroid cancer.

## 1. Introduction

Thyroid cancer is the most common endocrine cancer. In the United States, its incidence rate has been estimated at 12.2 cases per 100,000 individuals per year [1]. Thyroid carcinoma is the 9th most common cancer in women and the 18th cancer in both sexes, and it represents approximately 1% of all diagnosed cancers worldwide [2,3]. This cancer is classified into two histologic types: (i) follicular cell-derived cancers, such as papillary thyroid cancer (PTC; approximately 80% of all thyroid cancers), follicular thyroid cancer (FTC; 10% of all thyroid tumors), poorly differentiated thyroid cancer (4–6%), and anaplastic thyroid cancer (ATC, 2–5%) [4]; and (ii) parafollicular C cell-derived medullary thyroid carcinoma (MTC; 5–10% of all thyroid tumors) [5]. Multiple factors may play a role in their development. In breast tumors, mutations in tumor-suppressor genes (TSGs) have been identified as one of the important genetic mechanisms of breast carcinogenesis [6]. During carcinogenesis, gene alterations may affect the function of important players in normal cellular functions. These alterations may be gain-of-function mutations that particularly influence the activity of oncogenes, or loss-of-function mutations. Both may contribute to the development of the malignant phenotype [7]. TSGs play a regulatory role in cell proliferation by controlling cell-cycle progression (cell-cycle checkpoints) and consequently tissue cell proliferation. TSG deletions and other mutations may affect cancer cell proliferation, apoptosis, migration, invasion, and metastasis formation [8]. Moreover, TSGs are involved in cell differentiation regulation, genomic integrity maintenance, DNA damage repair, signaling pathways, and cell adhesion. Besides genetic alterations, other mechanisms are implicated in TSG inactivation, particularly the loss of expression due to epigenetic silencing or enhanced proteolysis of tumor-suppressor proteins [9]. These proteins are generally divided into five classes: intracellular proteins that modulate the cell cycle, hormone receptors that prevent cell proliferation, cell cycle-associated proteins that control checkpoints, apoptosis-promoting proteins, and DNA repair enzymes [10]. Studies on TSGs and their protein products are important because they may provide potential targets for thyroid cancer management. Better understanding these genes and their protein products can lead to new therapeutic applications. This review focuses on the more common TSG inactivation mechanisms and on the different altered forms of these genes in human thyroid cancer. It also discusses the current knowledge on therapeutic modalities for TSG targeting in thyroid cancer.

## 2. Major Classes of Tumor-Suppressor Genes

TSGs are classified into different categories on the basis of the function of their protein products. Caldas and Venkitaraman classified TSGs as “gatekeepers” or “caretakers” [11]. Recent reports suggested a third class of TSGs, called “landscapers” [12,13]. An exhaustive review of all known TSGs and their detailed functions is beyond the space available here. Therefore, we provide a list of these genes and the functions of their protein products (Table 1).

### 2.1. Gatekeepers

Gatekeeper genes encode proteins that regulate cell proliferation and tissue growth. These proteins are major components of the system that regulates and controls cell division and death [14]. They act as guardians to inhibit cell proliferation, promote apoptosis and other forms of programmed cell death, and facilitate cell differentiation [15,16]. Therefore, these TSGs are implicated in the regulation of the cell cycle, a highly controlled process in which two key regulators, called cyclins and cyclin-dependent kinases (CDKs), are involved [17]. Gatekeepers interact, directly or indirectly, with these regulators to control the cell cycle [18]. Mutations in these genes lead to a checkpoint bypass in normal cells, allowing for unlimited cell division, proliferation, growth, and transformation [19].

The gatekeeper concept was first proposed to describe the role of the adenomatous polyposis coli (APC) tumor-suppressor, which is mutated in the early stages of colorectal cancer [20]. The expression of wild-type APC in colorectal epithelial cells harboring *APC* mutations can lead to their apoptosis, suggesting that APC may regulate cell death [21]. Kay Macleod subdivided gatekeepers into three subclasses: “initiation gatekeepers”, “progression gatekeepers”, and “metastasis gatekeepers” [22]. This classification is based on the stage of tumorigenesis when specific TSG mutations are detected. For example, *APC* mutations are rate-limiting for colorectal tumor initiation, suggesting that APC is an “initiation gatekeeper”. On the other hand, p53 is a “progression gatekeeper” [22]. Macleod also proposed that gatekeeper TSGs are distinguished from caretakers and landscapers because their loss of function is a rate-limiting step in the multi-step tumorigenesis process. As they act directly to restrain tumor development, restoring their function inhibits neoplastic growth. 

Tumor protein p53 (*TP53*); retinoblastoma (*RB*); phosphatase and tensin homolog (*PTEN*); neurofibromatosis type 1 (*NF1*); and cyclin-dependent kinase inhibitor 2A, 2B (*CDKN2A*, *2B*) are other gatekeepers. *TP53* encodes the p53 protein, which is known as “the guardian of the genome” [23]. Rubbi and Milner also proposed a caretaker role for p53 because of its independent role in several DNA repair pathways [24]. However, mutant p53 has oncogenic functions that are associated with its ability to exert wild-type p53-independent oncogenic effects [25,26]. The protein encoded by the *RB* gene plays a pivotal role in cell-cycle control at the G1 checkpoint to block S-phase entry and cell growth [27]. The RB family includes three members (p105, p107, and p130) [28]. This protein represses the transcription of genes that are involved in the transition from the G1 to the S phase of the cell cycle by indirectly binding to their promoter, as a complex with E2F. Phosphorylated RB proteins can also repress gene transcription through interacting with chromatin remodeling proteins [29]. This suggests a gatekeeper function for phosphorylated RB in the regulation of cell proliferation and tissue growth. The *PTEN* gene encodes a phosphatase enzyme that is highly expressed in almost all body tissues [30]. This enzyme has an antagonizing effect on the phosphoinositol-3-kinase (PI3K)/AKT signaling pathway, leading to cell survival and cell proliferation inhibition. *PTEN* is the second most frequently mutated gene in human cancers after *TP53*. Various *PTEN* germline mutations have been reported in cancer susceptibility syndromes, such as Cowden syndrome, in which more than 80% of patients have inherited *PTEN* mutations [31]. The inhibition of PI3K/AKT signaling by PTEN results in the regulation of many cellular processes, such as survival, proliferation, energy metabolism, and cellular structures, suggesting a gatekeeper tumor-suppressor role [32].

*NF1* is another TSG with gatekeeper functions. This gene encodes a protein called neurofibromin that acts as a negative regulator of the *RAS* proto-oncogene, which plays a central role in cell-growth control [33]. *NF1* germline mutations cause a disorder called neurofibromatosis 1, characterized by changes in skin pigmentation and the development of different tumor types [34]. *NF1* seems to act both as a gatekeeper and landscaper tumor suppressor [22]. 

*CDKN2A* and *CDKN2B* encode three well-established tumor suppressors: p15, p16, and p14^ARF^. Both p15 (the product of *CDKN2B*) and p16 (the product of *CDKN2A*) bind to and inhibit CDK4 and CDK6 (18). In the absence of these two proteins, CDK4- and CDK6-dependent RB phosphorylation leads to its interaction with E2F and promotes cell growth [35]. P14^ARF^-specific interactions with E3 ubiquitin ligase MDM2 leads to its degradation and blocks its capacity to mask the transcriptional activating function of p53, which ultimately prevents p53-dependent oncogenic alterations [36].

### 2.2. Caretakers

Caretaker TSGs encode proteins that are involved in genome stability maintenance by decreasing the mutation rate of gatekeepers and oncogenes [13]. The term ‘genomic instability’ describes the many different variations observed in the genome, such as telomere damage, centrosome amplification, epigenetic changes, DNA damage, and chromosomal rearrangements [37,38]. Caretakers do not directly regulate cell proliferation and growth but hinder the accumulation of DNA damage and mutations within important exonic regions of the genome [39]. Thus, mutations affecting these genes promote cell transformation. Caretaker genes encode proteins that prevent/limit genomic instability by regulating different signaling pathways [40]. The function of caretaker TSGs in protecting the genome from mutations could help to inhibit cancer development. These tumor-suppressors commonly act by limiting DNA damage and/or by regulating DNA repair. Interestingly, these genes are also known as longevity-assurance genes because they inhibit or delay the development of aging phenotypes and age-associated diseases [41].

Deletions may cause the loss of caretaker TSG activity, thus promoting tumor aggressivity. Indeed, tumors with unstable genomes are more aggressive and more likely to recur after treatment, suggesting that caretakers play a pivotal role in harnessing tumor aggressiveness [42]. Breast cancer susceptibility gene 1 and 2 (*BRCA1* and *BRCA2*) are the best known caretakers [43] that play an important role in DNA repair. Their mutation results in cumulative mutations in cells and in cancer development. These genes encode two nuclear phosphoproteins that bind to RAD51 and MRE11, the two proteins that regulate DNA double strand break (DSB) repair and preserve chromosomal integrity [44,45]. Several studies have shown that cells harboring *BRCA1* and *BRCA2* mutations tend to accumulate chromosome abnormalities (including chromosomal breaks), abnormal mitoses, and aneuploidy [46,47]. BRCA1 participates in the regulation of the G1-S and G2-M transition checkpoints [48]. As mutations of these two genes increase the risks of breast and ovarian cancer [49], *BRCA1/2* mutation screening is recommended in women with a family history of disease-causing *BRCA* mutations [50]. Ataxia Telangiectasia mutated (*ATM*) is another well-known caretaker gene encoding a protein that phosphorylates BRCA1, leading to the activation of the G2-M checkpoint [51]. Ataxia-telangiectasia and rad3-related (*ATR*) and checkpoint kinase 2 (*CHEK2*), two other putative caretakers, are required for BRCA1 phosphorylation by ATM [51]. ATM plays a key role in genotoxic responses other than the DNA DSB response. For example, ATM activates the pentose phosphate pathway, the main source of the key antioxidant agent NADPH, in response to genotoxic stress [52].

### 2.3. Landscapers

The third group of TSGs is known as landscapers. When mutated, these genes contribute to tumor growth by creating a favorable microenvironment for uncontrolled cell proliferation [12]. It has been proposed that the mechanisms of action of landscaper genes involve the direct or indirect regulation of extracellular matrix proteins, cell-surface markers, adhesion molecules, and growth factors [22]. By acting on adjacent stromal cells, mutated landscapers indirectly create an abnormal microenvironment that promotes the transformation of epithelial cells [53]. This phenomenon was first described in a study that assessed mutations in juvenile polyposis syndrome and ulcerative colitis in which the neoplastic transformation of stromal cells surrounding the tumor leads to the creation of an abnormal stromal environment that affects the development and growth of epithelial cells [53]. However, Woodford-Richens et al. suggested that the theory involving the “crosstalk” between normal epithelium and abnormal stroma does not fully explain the subsequent transformation of epithelial cells [54].

Volmer et al. described the landscaping activity of the *SMAD4* gene in cultured cells by differential secretome analysis [55]. *SMAD4* encodes a protein that is a mediator of the transforming growth factor beta (TGF-β) signaling pathway [56]. Other known landscapers include the gene encoding E-cadherin, as well as *NF1*, *RB*, and *APC* [22,57]. Therefore, some TSGs play a variety of roles as tumor-suppressors and can be categorized into two or three subclasses.

## 3. Mechanisms of TSG Inactivation

Mutations (e.g., deletions, insertions, nonsense or missense mutations, and frameshift mutations) may lead to TSG inactivation or a loss of function and consequently to tumor growth [58]. To explain the underlying mechanisms, in 1971, Knudson proposed the two-hit hypothesis based on epidemiological data from patients with retinoblastoma, a type of retinal cancer [59]. He divided patients into two groups: (i) patients with family history of retinoblastoma who generally develop the tumor at an early age, show metastases, and often present tumors in both eyes with multifocal lesions in each eye [60]; and (ii) patients without family history (sporadic cancer) who are older at disease diagnosis and present more limited tumors, often only in one eye. To explain these differences, Knudson hypothesized that retinoblastoma develops as the result of two separate mutations in the two *RB1* alleles [60]. Patients with a family history of retinoblastoma inherit one copy of the mutated allele; thus, they receive the first “blow” before birth. Upon the occurrence of a somatic mutation (the second “blow”) on the other *RB1* allele, they will develop retinoblastoma. In the case of sporadic or non-hereditary retinoblastoma, both hits (mutations) should occur during the person’s life. This implies that *RB1* mutations in both alleles are associated with a loss of function [59,60]. Upon occurrence of the first mutation in one allele, the protein level will be reduced [61]. This may increase the rate of cell proliferation and facilitate cell transformation. The second mutation, which is more frequent than the first mutation, causes the inactivation of the second allele [61,62]. In recent years, much evidence has shown that this theory cannot be generalized. Significant data indicate that for many TSGs (e.g., *TP53*, *PTEN*), mutations in only one allele are sufficient to affect protein synthesis and may promote tumorigenesis. This phenomenon is called haploinsufficiency. In this case, one inherited mutation in one allele is sufficient to induce tumorigenesis [63].

In the last two decades, many findings have shown that X chromosome inactivation plays a role in the loss of heterozygosity (LOH; the loss of one of the parental alleles in a genetic locus) for X-linked TSGs [64]. In 1991, Klein et al. described for the first time the role of X-linked TSGs in cancer [65], followed by other studies [66,67]. The inactivation of X-linked TSGs in human cancer is not clearly understood, and the underlying mechanisms are different in men and women [68]. LOH in the X chromosome can promote tumor development and progression and is an example of a single genetic hit that leads to TSG loss of function, contrary to Knudson’s hypothesis [69].

**Figure 1:**
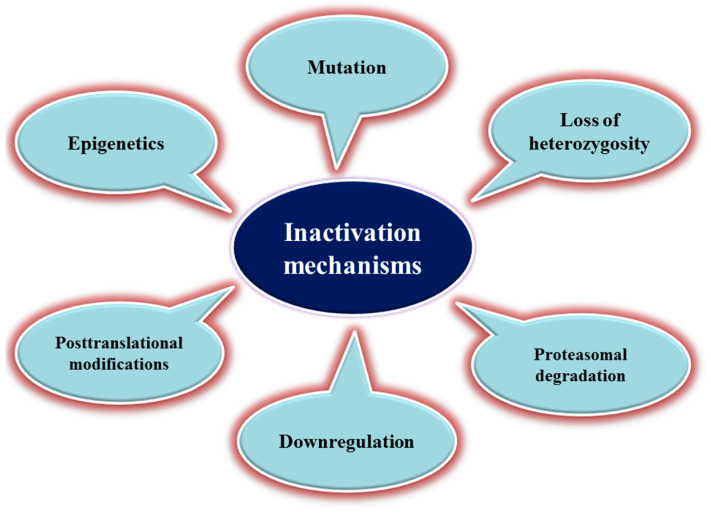
The different mechanisms of tumor-suppressor gene inactivation.

Promoter methylation also regulates gene expression. In some cases, TSG promoter methylation alteration may lead to gene silencing and a loss of function, without the need for gene mutations [70]. Other epigenetic mechanisms have been implicated in TSG inactivation, particularly microRNAs that silence these genes in various cancer types [71]. Post-translational modifications are other inactivating mechanisms observed in proteins encoded by TSGs. For example, PTEN phosphorylation at some specific residues represses its activity [72]. This tumor suppressor may also be inhibited through the oxidation of a cysteine residue in the active site of PTEN [73]. Figure 1 shows some common mechanisms of TSG inactivation in cancer.

## 4. Altered Tumor-Suppressor Genes in Thyroid Cancer

### 4.1. TP53

The loss of p53 function has been described in thyroid cancer. The majority of *TP53* gene mutations are within exons 5–8 [74]. *TP53* mutations are more frequent in ATC (50–80%) [75]. Moreover, the inactivation of p53 due to mutations or any other inhibitory mechanism may result in the progression from well-differentiated thyroid *cancer* to ATC [76,77]. This suggests that *TP53* mutations may be a late event in thyroid cancer. In addition, some studies have shown that increased p53 protein levels are associated with thyroid cancer. Immunohistochemistry analysis revealed higher p53 protein levels in anaplastic, poorly differentiated, and well-differentiated thyroid cancer samples [76,77,78,79]. 

Some studies found an association between p53 and some factors involved in signaling pathways that regulate p53 expression and promote thyroid cancers. For instance, murine double minute (MDM) family members are major regulators of the p53 expression level through its ubiquitination and proteasomal degradation [80]. Prodosmo et al. showed that in PTC, the overexpression of MDM4-S, a MDM4 variant; the expression of MDM4-211, an abnormal variant; or reduced MDM4 levels all lead to p53 inactivation [81]. HECT-, UBA-, and WWE-domain-containing E3 ubiquitin protein ligase 1 (HUWE1) is an ubiquitin E3 ligase for MDM2 that can regulate p53 stabilization. In fact, HUWE1 and MDM2 are part of a network of E3 ubiquitin ligases to regulate their substrates (e.g., p53) [82]. A study of some thyroid cancer cell lines to assess HUWE1 function demonstrated that HUWE1 downregulation leads to MDM2 overexpression and decreased p53 protein stability, suggesting that it may act as a TSG [83]. In addition, other factors play a role in p53 regulation, such as wild-type p53-induced phosphatase 1 (WIP1), the overexpression of which can inhibit p53 in PTCs [84]. In an animal study, Zou et al. found that *TP53* downregulation leads to higher levels of thyroid-stimulating hormone (TSH) and the subsequent upregulation of the PI3K/AKT pathway. This was associated with PTC-to-ATC transformation and ATC progression [85]. Altogether, the data mentioned above suggest a dual function of *TP53* as an oncogene and TSG.

### 4.2. PTEN

*PTEN* is a TSG associated with the negative regulation of signaling pathways, such as the PI3K signaling cascade [86]. The loss of PTEN activity in Cowden syndrome increases the risk of some cancers, including thyroid cancer [87]. Patients with this syndrome are at a higher risk of developing FTC due to pathogenic mutations in the *PTEN* gene [88]. PTEN is considered a predictive marker in patients with thyroid cancer and Cowden-like syndrome. For instance, Ngeow et al. showed that very low serum levels of PTEN in these patients could predict the presence of a germline *PTEN* mutation [89]. PTEN inactivation might be the result of different mechanisms, such as point mutations, deletions, promoter hypermethylation, and post-translational modifications [90,91,92,93]. *PTEN* somatic deletions and LOH have also been described in many tumor types, especially in thyroid cancer subtypes [94].

PTEN hamartoma tumor syndrome (PHTS) is a complex disease caused by germline *PTEN* gene mutations. These patients usually develop various benign and malignant tumors in different tissues, such as breast, thyroid, intestine, and skin [95]. The lifetime risk of thyroid cancer in patients with PHTS who have a PTEN mutation has been estimated at 35.2% [96].

Moreover, Nagy et al. found that 3–10% of patients with *PTEN* mutations have differentiated thyroid cancer (DTC) [97]. Specifically, in a sample of patients with DTC, 4.8% of patients with FTC harbored germline *PTEN* mutations, but none of the patients with PTC did [97]. Other studies also reported the presence of *PTEN* mutations in FTC tissue samples [98,99]. However, sporadic thyroid cancers do not harbor somatic *PTEN* mutations [100].

*PTEN* is also regulated through various epigenetic mechanisms. For example, aberrant *PTEN* methylation impairs PTEN function, leading to enhanced PI3K/AKT signaling, and thyroid cancer growth, progression, and metastasis formation [101]. Methylation-specific polymerase-chain reaction analysis of 59 thyroid cancer samples showed that the *PTEN* promoter was hypermethylated in 45.7% of PTCs, 85% of FTCs, and 83% of follicular adenomas [102]. Pringle et al. developed mouse models in which two TSGs, protein kinase CAMP-dependent type 1 regulatory subunit alpha (*Prkar1a*) and *Pten*, were concomitantly knocked out in the thyroid. They found that this double knockout led to FTC development with metastatic spread, and enhanced function of protein kinase A (PKA) and mammalian target of rapamycin (mTOR) signaling [103]. Some studies suggested that the loss of *PTEN* expression is associated with the low expression of p27, a cell-cycle inhibitor, in FTC and ATC specimens [104,105].

Ubiquitination also might be implicated in PTEN inhibition. Yu et al. found that two succinate dehydrogenase-D variants, G12S and H50R, induce PTEN mono-ubiquitination, leading to its translocation into the nucleus where PTEN causes AKT upregulation and promotes tumorigenesis via FOXO3a phosphorylation and autophagy downregulation [106]. Frisk et al. observed no relationship between *PTEN* expression, LOH, and mutation status in the ATC samples under study. They suggested that PTEN inactivation may be due to a number of epigenetic and/or structural silencing mechanisms rather than to classical biallelic inactivation [107].

### 4.3. APC

Germline mutations in the *APC* gene lead to familial adenomatous polyposis (FAP) syndrome [108]. Most *APC* mutations are nonsense and frameshift mutations [109]. In 1949, Crail described for the first time the development of thyroid cancer in patients with FAP [110]. Based on the Leeds Castle Polyposis Group database, the incidence of thyroid cancer in patients with FAP is 1.2% [111]. The mean age at diagnosis of thyroid cancer in these patients is between 25 and 28 years [111,112]. PTC frequency in patients with FAP is higher in women than in men (10–17:1) [113,114].

*APC* mutations are rarely detected in sporadic thyroid cancers and may play a role in thyroid cancer development [115]. Moreover, *APC* mutations are associated with *RET/PTC1* gene rearrangements in patients with FAP and thyroid cancer [116]. *APC* gene mutation analysis in a 25-year-old woman with FAP and previous total colectomy and thyroidectomy revealed the presence of a germline mutation in exon 13 [117]. In this study, exons 1–15 of the *APC* gene and exon 3 of the beta-catenin gene were analyzed in genomic DNA extracted from peripheral blood samples and 12 cribriform-morular variants of papillary thyroid carcinoma nodules. Besides the germ-line mutation, six somatic mutations between codons 308 and 935 of the *APC* gene were found, but none were found in the beta-catenin gene [117]. Cetta et al. reported that 13/15 patients with FAP and thyroid cancer carried *APC* mutations between codons 778 and 1309 [118]. Truta et al. analyzed *APC* in 14 patients with FAP and PTC and identified germline mutations (located before codon 1286 and outside the *APC* mutation cluster region) in 12 patients [119]. Another study showed that the risk of developing thyroid cancer is higher in individuals with *APC* mutations at the 5’ end, near codon 528, and that this risk further increases in subjects harboring a mutation at codon 1061 [120]. Han et al. carried out an *APC* mutation screening in Korean patients with FAP and identified nine truncating mutations, one missense mutation, seven polymorphisms, and three intronic variants [121]. In a European cooperative study, the analysis of *APC* mutations in 15 women with FAP and PTC uncovered *APC* germline mutations in 13 of them at codons 140, 593, 778, 976, 993, 1061 (*n* = 5), 1105 (*n* = 1), and 1309 (*n* = 2) [112].

Some data indicate that epigenetic mechanisms also are implicated in the regulation of APC expression. Mir-155 can target *APC* by direct binding to its 3’-untranslated region, and this may decrease APC mRNA and protein levels [122]. Zhang et al. found that upregulation of mir-155 results in *APC* downregulation and the activation of WNT/β-catenin signaling, which in turn promotes PTC cell growth, viability, and colony formation. This suggests that mir-155 may play an oncogenic role in these tumors [122]. APC protein has a binding site for β-catenin that consists of seven 20-amino acid repeats (20-AARs) [123]. Kumamoto et al. carried out a retrospective study to find an association between the number of 20-A ARs and thyroid cancer development [124]. They observed that in three patients with FAP and PTC, one patient had only two 20-AARs in the germline *APC* mutation and none in the somatic *APC* mutation, and the other two did not have any remaining 20-AAR. Moreover, in 13/16 patients with FAP and thyroid cancer (81.3%), the remaining number of 20-AARs was zero. Therefore, they suggested that in patients with FAP and thyroid cancer, the APC/β-catenin signaling pathway may play an important role in the pathogenesis of this cancer [124].

### 4.4. RASAL1

RAS protein activator like 1 (*RASAL1*) is the negative modulator of the RAS signaling pathway that has been identified as a key TSG in thyroid carcinoma [125]. The RAS-coupled mitogen-activated protein kinase (MAPK) and PI3K pathways play pivotal roles in thyroid cancer development. Therefore, abnormal *RASAL1* gene expression may affect these pathways and thyroid cancer development [126]. In an in vitro and in vivo study, Lui et al. investigated alterations of genes encoding negative modulators of the RAS-coupled MAPK and PI3K pathways in thyroid cancer. They discovered *RASAL1* gene-disabling mutations and promoter hypermethylation in thyroid cancer samples, predominantly FTC and ATC, suggesting the *RASAL1* is a key TSG in thyroid cancer. They found one nonsense and six missense mutations that were located at highly conserved sites within the RAS GTPase activating domain of RASAL1 [126].

Ngeow et al. investigated the presence of germline *RASAL1* mutations and *PTEN* mutation status in patients with Cowden syndrome and thyroid cancer. Among the included 155 patients, they identified deleterious *RASAL1* germline mutations in two patients with wild-type *PTEN* who had FTC. They also detected detrimental germline *RASAL1* mutations in patients with Cowden syndrome and follicular-variant PTC, unlike many other patients with Cowden syndrome [127]. Charalampos et al. described a patient with MTC, mesothelioma, and meningioma who harbored *APC* and *RASAL1* mutations based on whole exome sequencing (WES) data. This indicates a possible TSG role for both *APC* and *RASAL1* in thyroid cancer development [128]. Moreover, WES data indicate the presence of *APC* and *RASAL1* gene alterations in various thyroid cancer subtypes. For example, WES and Sanger sequencing of a MTC sample from a 57-year-old woman with sporadic MTC showed two germline *APC* and *RASAL1* variants [129]. Moreover, the targeted exome sequencing of DNA from 11 formalin-fixed, paraffin-embedded ATC tissue samples uncovered two specimens (18%) with a *RASAL1* mutation that was significantly associated with shorter survival [130].

### 4.5. TP63 and TP73

Several studies found a possible role for p63, a p53 homolog, in thyroid cancer development [131,132]. *TP63* is expressed in epithelia of ectodermal origin. This gene has six different isoforms with a transactivating effect or dominant negative activity on p53 target genes [131]. Bonzanini et al. reported p63 immunostaining in PTC samples but not in controls [133]. In another study, *TAp63α*, an isoform of the *TP63* gene, was detected in thyroid cancer samples. The authors suggested that it may act as an oncogene by promoting thyroid cancer progression via the disruption of p53 tumor-suppressor activity [134]. However, *TAp63β* and *TAp63γ* (two other p63 isoforms) have tumor-suppressor activity in PTC and FTC cells [134]. These data show that *TP63*, like *TP53*, may play a dual role in thyroid cancer: oncogene and TSG. P73 is another member of the p53 family that plays a controversial role in thyroid cancer. Ferru et al. reported that the expression of *TAp73* and *DNp73*, two p73 isoforms, is reduced in follicular adenomas, FTCs, and PTCs. The TAp73 variant has pro-apoptotic, and DNp73 has anti-apoptotic, properties [135].

Some immunohistochemical studies revealed that p73 and DNp73 are expressed in human thyroid cancer specimens. These results were also confirmed by RT-PCR analysis showing that DNp73a is expressed in malignant thyroid tissues but not in normal tissues [136,137]. Periostin is a mesenchyme-specific protein that, when overexpressed, is associated with aggressive forms of thyroid cancer [138]. Puppin et al. suggested that DNp73α could induce periostin gene expression in papillary, follicular, and undifferentiated thyroid cancer cells [139]. Vella et al. showed that DNp73α overexpression in thyroid cancer cells leads to decreased PTEN expression [140]. Conversely, Malaguarnera et al. suggested that in thyroid cancer cells, TAp73α promotes p53 protein expression by inhibiting MDM2-mediated p53 degradation, proposing a thyroid-specific dual function for this TSG [134].

### 4.6. RB

Dysregulated *RB* expression in thyroid cancer has been demonstrated in several previous studies [141,142,143]. *RB* mutations and other inactivating mechanisms may play a role in thyroid cancer pathogenesis, especially MTC [141]. The cyclin-dependent-kinase inhibitor (CDKI)-RB1 pathway plays a pivotal role in the control of cell-cycle checkpoints. Loss of CDKIs leads to increased RB phosphorylation and consequently uncontrolled cell-cycle progression. Some studies showed the impairment of this pathway, which can promote MTC tumorigenesis [144,145]. In a conditional mouse model, Pozo et al. found that CDK5 overactivation in C-cells promotes sporadic forms of MTC through *Rb* downregulation [146]. Gilbert et al. observed that the inactivation of RB1 regulatory pathways in parafollicular C-cells leads to a high rate of MTC in mouse models [145]. Similarly, *Rb1* deletion may induce MTC in mice [147]. By immunohistochemistry, Valenciaga et al. demonstrated that RB expression reduction is associated with aggressive MTC [148]. More interestingly, the loss of RB expression in PTC samples has been correlated with aggressive early-metastasizing forms of this thyroid cancer [149].

### 4.7. PRKAR1A

The *PRKAR1A* gene encodes the regulatory subunit 1 alpha of PKA and is involved in the PKA signaling pathway. The binding of ligands to their receptors at the cell membrane could cause the activation of G-proteins that then induce adenylyl cyclase enzyme activity and convert AMP to cyclic AMP (cAMP). Upon cAMP binding to regulatory subunits, the PKA enzyme is activated and phosphorylates serine and threonine residues on substrate proteins. The cAMP response element (CRE)-binding protein (CREBP) is a transcription factor that is phosphorylated by PKA and translocated into the nucleus, where it binds to CREs and upregulates the expression of different genes. Through this kinase activity, PKA could regulate different cellular processes, including growth, division, and differentiation. TSH is one of the ligands that could activate PKA via its receptor. Elevated TSH could contribute to the development of thyroid cancer [150]. On the other hand, the phosphatase PTEN could dephosphorylate the CREBP transcription factor in the nucleus [151]. Zhang and colleagues showed that the inhibition of phosphodiesterase 4, which is responsible for cAMP degradation, could lead to *TP53* upregulation, followed by cancer cell proliferation inhibition and apoptosis induction [152]. PKA induction (via *PRKAR1A* downregulation), through adrenaline receptor activation, and *CHEK2* downregulation by the glucocorticoid receptor, could inhibit the DNA damage response, leading to the dysregulation of the DNA repair system; apoptosis; and cell-cycle checkpoint deregulation and carcinogenesis [153].

The Carney complex is a familial neoplasia syndrome associated with thyroid tumors. *PRKAR1A* is often mutated in this syndrome [154]. Moreover, LOH for the *PRKAR1A* locus has been reported in undifferentiated thyroid cancers (ATC) [155]. Pringle et al. showed that *Prkar1a* knockout in the thyroid can lead to hyperthyroidism and FTC development in mice [156]. They also developed mice in which both *PRKAR1A* and *PTEN* are knocked out and found that these animals develop FTC with a metastatic phenotype [103].

### 4.8. CHEK2

*CHEK2* is one of the most important genes involved in cell-cycle control by encoding the human analog of the yeast checkpoint kinases Cds1 and Rad53 [157]. CHEK2 acts as a protective regulator of cell division in response to DNA damage by preventing cell entry into mitosis. This suggests that *CHEK2* might be a TSG. Abnormalities in cell proliferation and apoptotic evasion due to *CHEK2* gene mutations have been observed in various cancers [158,159,160]. In a study on the association of *CHEK2* mutations with different cancer types in Poland, the most significant correlation was observed between thyroid cancer (mainly PTC) and *CHEK2* protein-truncating mutations [161]. Ten years later, Wójcicka et al. investigated deleterious polymorphisms in ATM, CHEK2, and BRCA1 in 1,781 patients with PTC and 2081 healthy controls using the Sequenom technology. They found that the *CHEK2* rs17879961 variant is associated with increased PTC risk [162]. However, Fayaz et al. analyzed the two most common *CHEK2* gene mutations in 100 DTC samples and found that they are not associated with higher DTC risk in the Iranian population [163]. This suggests that these two mutations are not detrimental and do not inactivate CHEK2. Kaczmarek-Ryś et al. used a genotyping technique to identify specific *CHEK2* alterations in 602 patients with DTC and 829 controls [164]. They found that the *CHEK2* c.470C variant increases the risk of DTC in the Polish population. Another genetic study to estimate the somatic alteration profile in FTC samples using next-generation sequencing identified *CHEK2* gene alterations in FTC samples [165].

**Figure 2:**
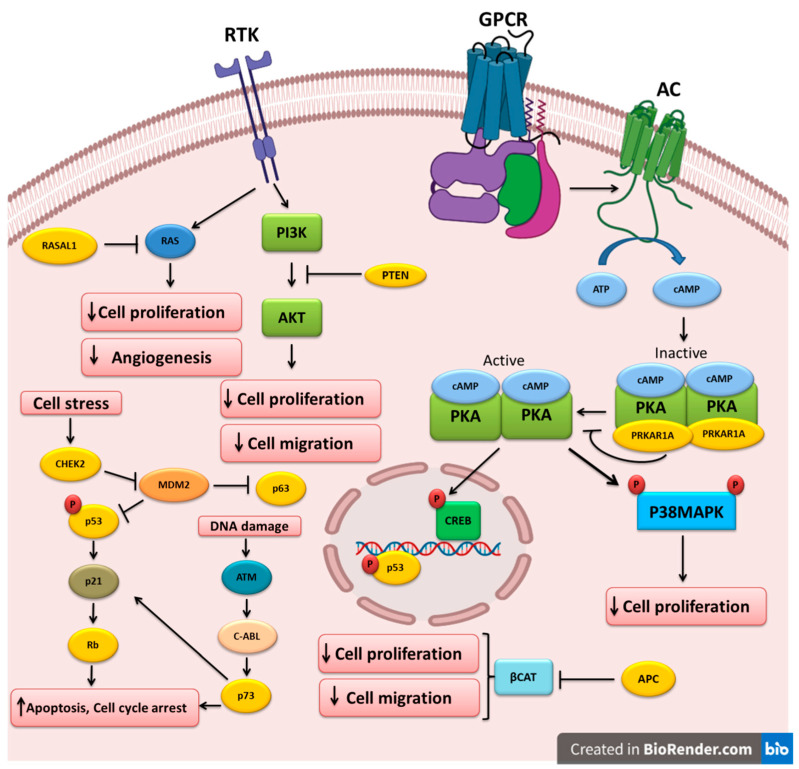
Key tumor-suppressor genes and their related pathways in thyroid cancer. Growth factors bind to their receptor tyrosine kinases (RTK) and activate the PI3K and RAS pathways that could be inhibited by PTEN and RASAL1, respectively. Cell stress triggers CHEK2, which in turn downregulates MDM2, leading to the upregulation of the p53 and p63 tumor-suppressors. P53 can also increase RB expression. DNA damage could lead to p73 upregulation and apoptosis induction. APC inhibits beta-catenin, leading to the inhibition of cell proliferation and migration. Ligand binding to G-protein-coupled receptors (GPCR) induces G-protein and adenylyl cyclase (AC), which increases cAMP and PKA activation. *PRKAR1A* mutations could cause PKA hyperactivation and cell proliferation induction.

The different TSGs altered and implicated in the pathogenesis of thyroid cancer are shown in Table 2. The molecular pathways that are regulated by these TSGs are depicted in Figure 2.

## 5. Clinical Significance and Therapeutic Potential

Recently, several therapeutic approaches for targeting TSGs have been developed. Although there are several promising strategies to restore TSG function, the most efficient modalities are those that act by controlling TSGs, thus inhibiting aberrantly stimulated signaling pathways, and that exploit the effects of TSG loss on cancer cells [43]. Thyroid cancer represents a good target for gene therapy strategies because (i) target genes can be specifically expressed in tumor cells using thyroid-specific promoters, thereby decreasing extra-tumoral toxicity; and (ii) patients with this cancer can receive complete thyroid hormone replacement therapy [166]. For example, a tissue-specific recombinant adeno-associated virus (rAAV) vector has been developed for targeted gene therapy of MTC with remarkable effectiveness both in vitro and in vivo. This rAAV vector contains a modified calcitonin gene promoter that specifically induces gene expression in calcitonin-secreting parafollicular C-cells of MTC tumors [167]. The reintroduction of wild-type p53 restores different biological processes and inhibits pathological events in human thyroid cancer cells. For example, transfection of the human ARO cell line (ATC-derived), which harbors mutated p53, with wild-type p53 significantly inhibits the proliferation rate and increases the percentage of cells arrested in the G0/G1 phase of the cell cycle. This effect was accompanied by a significant impairment in the tumorigenic potential of these cells and increased responsivity to TSH through upregulation of thyroglobulin, thyroid peroxidase, and the TSH receptor [168]. However, Fegin et al. reported that the transfection of wild-type p53 in clonal undifferentiated thyroid carcinoma cell lines harboring mutated p53 does not allow one to obtain many stably transfected cell clones. Indeed, they obtained only one clone that expressed wild-type p53 and that again expressed thyroid peroxidase. Nevertheless, they suggested that p53 plays a significant role in the determination or maintenance of cell differentiation in thyroid neoplasms [169]. It has been shown that p53 induces immune responses to suppress tumor development. In agreement, Zeki et al. transfected a p53-deficient thyroid cancer cell line (FRO) with wild-type *TP53* and found that p53 was implicated in the re-expression of the major-histocompatibility-complex (MHC)-class-II antigen, and consequently the MHC-class-II-associated stimulation of CD4+ cytotoxic T cells. Their results suggest that p53 loss in thyroid cancer cells could lead to low or no immune response against such tumors [170]. The immunohistochemical analysis of tissue sections from 206 DTCs, 105 benign nodules, and 18 normal tissues showed that the migration of CD8+ tumor-infiltrating lymphocytes is significantly higher in p53-positive than in p53-negative DTC specimens [171]. An in vivo study by Nagayama and colleagues on the role of p53 re-expression in thyroid cancer suppression using FRO cells (ATC) transfected with wild-type p53 highlighted the effect of this TSG on thyroid tumor-growth suppression. Specifically, wild-type p53 re-expression blocked tumorigenesis and induced a state of dormancy in these cancer cells. Moreover, more than 40% of mice xenografted with p53-transfected FRO cells were tumor-free, and the other animals developed tumors with very low vascularity compared with mice xenografted with the parental (p53-deficient) cell line. These results suggest that p53-based gene therapy can hamper thyroid tumor development and angiogenesis [172]. Wild-type p53 also regulates the expression of sodium iodide symporter (NIS). Liu et al. showed that wild-type p53 might increase the therapeutic effect of radioiodine by regulating NIS expression in the human ATC cell line 8505c. Specifically, they found that the expression of wild-type p53 in these cells transactivates the NIS promoter and improves radioiodine uptake, leading to the induction of apoptotic cell death compared with the parental cell line harboring mutated p53 [173]. 

Narimatsu et al. transfected p53-deficient FRO cells (ATC-derived) with a temperature-sensitive plasmid to stably express a p53 mutant (p53Val138). They observed lower proliferation rate in transfected cells compared with parental cells when they were cultured at 32 °C (permissive temperature) but similar rates at 37 °C (non-permissive temperature). Transfected cells also displayed a significantly higher sensitivity to radiotherapy. However, thrombospondin-1 (an antiangiogenic factor) expression was decreased, and vascular endothelial growth factor (an angiogenic factor) expression was similar to in parental cells. This implies that p53 restoration does not inhibit angiogenesis in this ATC-derived cell line. Nevertheless, these data are indicative of the interest of combining p53 gene therapy and radiotherapy for ATC treatment [174]. Imanishi et al. assessed the effects of p53 gene therapy combined with the histone deacetylase inhibitor depsipeptide in two ATC cell lines, one with very low p53 expression and the other without p53 expression. They concluded that depsipeptide enhances the apoptosis-inducing effects of p53 [175]. Moretti et al. showed that in thyroid cancer cells, p53 is efficiently transduced only in cells harboring a mutated *TP53* gene, while it is ineffective in wild-type *TP53*-expressing cells [176]. It appears that apoptosis is the major mechanism of cell killing by *TP53* gene therapy in ATC cells [177]. Gene therapy approaches have been also evaluated in PTC cells. For example, the injection of p53-transfected NPA cells (PTC-derived) in nude mice resulted in the inhibition of tumor growth in a dose-dependent manner [178]. MTC has been considered a good target for gene therapy because of the possibility of direct and specific gene expression using vectors with a modified calcitonin promoter and the availability of animal models of this tumor. Therefore, future gene therapy approaches can be easily tested using these tools [179].

Furthermore, a study showed that the small compound PRIMA-1 (p53 reactivation and induction of massive apoptosis) restores the p53-HSP90α interaction, improves the translocation of this complex into the nucleus of tumor cells, and leads to the activation of p53 target genes [180]. A study on the mechanism of PRIMA-1-dependent rescue of mutant p53 function in thyroid cancer cells showed that PRIMA-1 stimulates DNA demethylation in cells harboring mutant p53, mainly by suppressing the expression of the DNA methyltransferases 1, 3a, and 3b and by increasing the expression of growth arrest and DNA damage-inducible α (GADD45α) and ten-eleven translocation (TET) family members [181]. In addition, in vitro studies revealed that PRIMA-1 decreases the viability of thyroid cancer cells harboring p53 mutations and enhances the cytotoxic effects of doxorubicin and cisplatin in chemotherapy-resistant thyroid cancer cells [182]. The combination of BKM120 (PI3K inhibitor) with PRIMA-1 synergistically hampers the growth of thyroid cancer cells in culture and after injection in mice by inhibiting the PI3K/AKT/mTOR and CPSF4/hTERT pathways and reactivating mutant p53 [183]. 

Zn (II)-curc is a small curcumin-based zinc compound that downregulates mutated p53 and reactivates the functions of wild-type p53, leading to the transactivation of p53-target genes [184]. The upregulation of MDM2, a p53 suppressor, alters the balance between MDM2 and p53 and promotes tumor initiation. Restoring p53 function by hindering its interaction with MDM2 also is an interesting therapeutic approach for the treatment of dedifferentiated PTCs that express wild-type p53. For instance, APG115, an antagonist of the MDM2-p53 interaction, increases p53 activity, and this leads to decreased dedifferentiated PTC cell viability, apoptosis induction, and tumor regression in a xenograft mouse model [185]. Strategies to increase PTEN expression also have been developed. DNMT1 expression inhibition using shikonin (a plant secondary metabolite) increases PTEN protein expression, leading to the suppression of PTC cell migration [186]. Exposure of FRO cells (ATC-derived line) to carboplatin combined with radachlorin-photodynamic therapy inhibits the expression of EGFR and PI3K and activates PTEN, which prevents tumor growth and stimulates apoptosis [187]. Weng and colleagues reported that ectopic *PTEN* expression in thyroid cancer cell lines, in which PTEN protein expression is low due to mRNA downregulation, could contribute to cell arrest in the G1 phase and apoptosis induction [188]. Moreover, *TP73* gene inhibition using a small interfering RNA could reduce p53 transcriptional activity in thyroid cancer cells. Interestingly, ectopic co-expression of transcriptionally active isoforms of *TP73* (TAp73α) and *TP53* in thyroid cancer cells increases their tumor-suppressor activity compared with single expression of TP53 or TAp73α. The MDM2-dependent effect of TAp73α on p53 upregulation is due to the antagonizing impact of p53 on MDM2 induction. Based on these findings, the use of a mutant form of p73 (TAp73α-G264W) could be interesting to induce p53 tumor-suppressor activity in thyroid cancer cells [189]. Unfortunately, no clinical data on TSG targeting for thyroid cancer management is available yet.

## 6. Future Directions

To date, no clinical data are available on the efficacy and safety of TSG therapy for thyroid cancer management. The current knowledge on TSG-based therapy for this cancer is mainly based on preclinical studies. Thus, it is necessary to set up clinical studies with a large number of participants to accurately evaluate the effectiveness and safety profiles of TSG-based therapy for thyroid cancer. Moreover, comprehensive studies with large sample sizes should be carried out to identify other TSG gene alterations and pathological mutations in thyroid cancer using next-generation sequencing. The data acquired by this method can be used for screening, diagnosis, and prognosis purposes, leading to better clinical decision-making and the early management of patients with thyroid cancer.

## 7. Conclusions

TSG expression and function can be directly and indirectly inhibited through several mechanisms. Understanding TSG inactivation mechanisms and the signaling pathways aberrantly activated upon TSG inhibition in pathological conditions expands our knowledge on thyroid cancer initiation and progression and can help to develop novel targeted therapies. To date, several altered TSGs related to thyroid cancer have been reported, as listed in this review. However, the current knowledge on the potential roles of TSGs and their alterations at various stages of thyroid cancer is not sufficient. A variety of sensitive and specific methodologies have been developed to identify alterations of these genes and their protein products. Taken together, all TSGs mentioned in this review article have the potential to become predictive, prognostic, diagnostic, and therapeutic markers for thyroid cancer.

## Figures and Tables

**Table 1:** The different subclasses of tumor-suppressor genes in thyroid cancer.

Major Classes	Examples	Main Functions
Gatekeepers (1) Initiation gatekeeper (2) Progression gatekeeper (3) Metastasis gatekeeper	*APC* *TP53*, *PTEN*, and *TP63* *PTEN* *PTEN*	The regulation of cell proliferation/division, tissue growth, and apoptosis
Caretakers	*ATM*, *XRCC3*, *XPC*, *ERCC5*, *ATR*, and *BRCA1*	The maintenance of genomic stability and DNA repair mechanisms
Landscapers	*SMAD4*, *CDH1*, *NF1*, *RB*, and *APC*	The regulation of extracellular matrix proteins, cell-surface markers, adhesion molecules, and growth factors

**Table 2:** Altered tumor-suppressor genes involved in thyroid cancer pathogenesis.

Tumor Suppressor Gene	Normal Function of Protein Product	Type of Alterations	Affected Thyroid Tumors	Mutation Frequency
*TP53*	Cell-cycle regulation	Point mutations, negative regulation by MDM family members, and ubiquitination. Dual function: oncogene and TSG	ATC (50–80%), PTC	40% in PTC, 60% in ATC
*PTEN*	Cell division regulation	Point mutations, deletion, promoter hypermethylation, LOH, ubiquitination, and post-translational modifications	FTC, DTC, ATC, and PTC	65–85%
*APC*	The regulation of cell division, adhesion, and migration	Nonsense and missense mutations, frameshift mutations, polymorphisms, and epigenetic regulation	ATC, MTC, PTC, FTC, and CMVPTC	87% in FAP-associated PTC
*RASAL1*	The stimulation of the GTPase activity of RAS	Missense and nonsense mutations, promoter hypermethylation	PTC, ATC, FTC, and MTC	17% in ATCs, 5% in FTCs, and 3% in PTCs
*TP63*	The regulation of cell proliferation and differentiation, the transactivating effect, or dominant negative activity on p53 target genes	Downregulation, dual function: oncogene and TSG	PTC and FTC	
*TP73*	Involved in cellular responses to stress and development	Downregulation and upregulation, dual function: oncogene and TSG	Follicular adenoma, FTC, and PTC	
*RB*	The control of DNA replication and cell division during cell damage	Mutations, deletions, downregulation, enhanced phosphorylation, and the loss of expression	MTC, PTC	1.8% in MTC
*PRKAR1A*	Promoting cell growth and division	Downregulation, LOH	FTC, ATC	LOH of the PRKAR1A(CA)n locus in 37.5% of cases
*CHEK2*	Cell-cycle control	Mutations, polymorphisms	PTC, FTC, and DTC	15.2%

PTC, papillary thyroid cancer; FTC, follicular thyroid cancer; ATC, anaplastic thyroid cancer; MTC, medullary thyroid carcinoma; DTC, differentiated thyroid carcinoma; CMVPTC, cribriform-morular variant of papillary thyroid carcinoma; and LOH, loss of heterozygosity.
